# Probing the origin of estrogen receptor alpha inhibition *via* large-scale QSAR study†

**DOI:** 10.1039/c7ra10979b

**Published:** 2018-03-27

**Authors:** Naravut Suvannang, Likit Preeyanon, Aijaz Ahmad Malik, Nalini Schaduangrat, Watshara Shoombuatong, Apilak Worachartcheewan, Tanawut Tantimongcolwat, Chanin Nantasenamat

**Affiliations:** Center of Data Mining and Biomedical Informatics, Faculty of Medical Technology, Mahidol University Bangkok 10700 Thailand chanin.nan@mahidol.edu +66 2 441 4371 ext. 2715 +66 2 441 4380; Department of Community Medical Technology, Faculty of Medical Technology, Mahidol University Bangkok 10700 Thailand; Center for Research and Innovation, Faculty of Medical Technology, Mahidol University Bangkok 10700 Thailand

## Abstract

Estrogen is an important component for the sustenance of normal physiological functions of the mammary glands, particularly for growth and differentiation. Approximately, two-thirds of breast cancers are positive for estrogen receptor (ERs), which is a predisposing factor for the growth of breast cancer cells. As such, ERα represents a lucrative therapeutic target for breast cancer that has attracted wide interest in the search for inhibitory agents. However, the conventional laboratory processes are cost- and time-consuming. Thus, it is highly desirable to develop alternative methods such as quantitative structure–activity relationship (QSAR) models for predicting ER-mediated endocrine agitation as to simplify their prioritization for future screening. In this study, we compiled and curated a large, non-redundant data set of 1231 compounds with ERα inhibitory activity (pIC_50_). Using comprehensive validation tests, it was clearly observed that the model utilizing the substructure count as descriptors, performed well considering two objectives: using less descriptors for model development and achieving high predictive performance (*R*_Tr_^2^ = 0.94, *Q*_CV_^2^ = 0.73, and *Q*_Ext_^2^ = 0.73). It is anticipated that our proposed QSAR model may become a useful high-throughput tool for identifying novel inhibitors against ERα.

## Abstract 2

This study compiles a large, non-redundant set of compounds tested for ERα inhibitory activity and applies QSAR modeling for unveiling the privileged substructures governing the activity.

## Graphical abstract

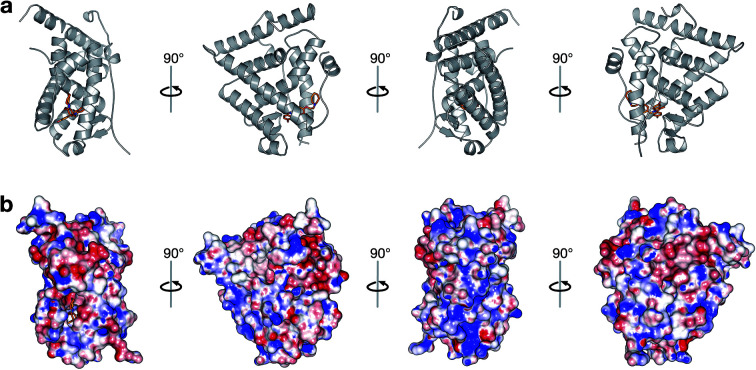

## 1 Introduction

Breast cancer is a serious public health concern worldwide^1^ with 14.1 million new cancer cases,^2^ accounting for an estimated death of 8.8 million in 2015.^3^ The global burden of breast cancer has increased as more than 1.7 million women are annually diagnosed with breast cancer.^4^ Out of all the cases, two-thirds of breast cancers are estrogen receptor (ERs) positive whereby the cancer cells consisting of ERs, when bound to estrogen, are signalled to proliferate.^5^ The metabolism of estrogen results in increased oxidative stress along with the production of genotoxic metabolites that form DNA adducts thereby causing genomic instability and eventually leading to the initiation of cancer.

**Fig. 1:**
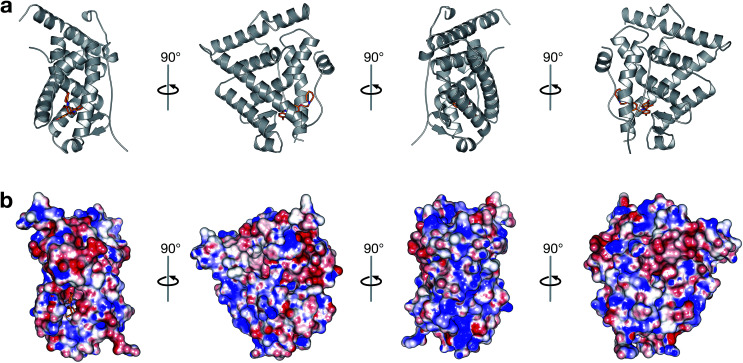
Crystal structure of ERα ligand-binding domain. The protein structure is shown as a cartoon depiction (a) while its electrostatic surface was rendered by APBS (b). The ligand is depicted as orange colored sticks for both panels; helices are colored gray for panel a; and surface is colored according to their electrostatic potential in which red and blue denotes negatively and positively charged surface patches, respectively, for panel b.

ER belongs to the steroid nuclear receptor superfamily and consists of two major subtypes namely, ERα and ERβ. The former is comprised of 595 residues and found on chromosome 6q while the latter is comprised of 530 residues and found on chromosome 14q. ERs have two major functional domains, the DNA-binding domain (DBD), which is responsible for DNA binding and dimerization, and the ligand-binding domain (LBD) that plays an important role in binding to different ligands and interacting with co-regulatory proteins. In addition, the N-terminus of ERs are highly viable and contain a transactivation domain, which interacts directly with other transcription factors. Furthermore, the C-terminus of the ERs are thought to affect the transactivation capacity of the receptors Fig. 1.^6^ Most ligands can bind to both types of ERs but differ in their binding affinities^7^ due to the high similarity of the ERα and ERβ in their DBD and a 55% similarly in their LBD.^8^ In response to estrogen, ERα and ERβ function as ligand-activated transcription factors that bind the estrogen response elements (EREs) and interact with co-activator or co-repressor proteins to regulate gene transcription.^9–12^ Aside from causing cancer, abnormal ER signaling may also give rise to cardiovascular, metabolic, inflammatory and neurodegenerative diseases as well as osteoporosis.^13^

Apart from the genomic effects, ERs are also known to exert extra-nuclear actions by which they regulate important cellular processes such as, leading cell proliferation, cell differentiation, and cell signaling to contributing to a biological outcome of tumor angiogenesis.^14,15^ Both ERα and ERβ are crucial for regulating mammary growth and development.^16,17^ Under normal physiological conditions, ERα mediates the proliferative actions of E2 which can be opposed by ERβ and together these receptors maintain a subtle balance of estrogen signalling in the cells.^18^ ERα is normally expressed in only 10–20% of human mammary epithelial cells while 80–85% of cells express ERβ.^19,20^ In contrast, the expression of ERα is increased while that of ERβ is decreased in breast cancer cells.^19,21^ Therefore, the expression of ERα is used as a measure of steroid hormone receptor status and is currently an acceptable prognostic marker for predicting the response to hormonal therapy.^22^ Unfortunately, the role of ERβ in breast cancer is still not well understood.^23,24^ However, it has been postulated that increased protein levels of ERβ are linked to a better prognosis, increased survival and a better response to anti-estrogen therapy.^24^

As previously mentioned, the expression of ERα is greatly increased in breast cancer cells and as such represents a promising therapeutic target for combating breast cancer. Anti-estrogens are agents that can hinder the production or utilization of estrogen and are categorized into two general classes: (i) selective estrogen receptor modulators (SERMs) and (ii) the so-called “pure” antagonists. The first class or SERMs are drugs that competitively binds ERα and ERβ and function by direct agonistic or antagonistic interactions. The outcome of such ER-binding is tissue-dependent meaning that some SERMs may exert agonistic response in one tissue and antagonistic response in another tissue. Tamoxifen represents a drug in the class of SERMs that serves as the first line of treatment against breast cancer. It is currently being administered to patients in an effort to regress tumor growth of ER positive (ER+) breast cancers. The second class of anti-estrogens or “pure” antagonists (*i.e.* ICI 182780 also known as Fulvestrant/Faslodex) works by preventing the binding of helix-12 to the surface of the ligand-binding domain, which in turn prevents the transcriptional activation of ERα. In spite of current endocrine therapies against estrogen, which represents a significant advance in breast cancer therapy in which many women develop resistance to current drugs. The selection and outgrowth of breast cancers resistant to endocrine therapy is common and most deaths arising from breast cancer are found in patients with ERα+ tumors.^25^ Moreover, in ERα+ breast cancers, one-third of women treated with tamoxifen for a period of 5 years will develop a recurrent disease within 15 years. Thus, the development of tamoxifen and aromatase inhibitor resistance remains a key problem in breast cancer treatment.^25^

Computational approaches have often been used to complement experimental studies for several reasons, which among others include: (i) handle and manage large volumes of biological and chemical information, (ii) model biomolecular phenomenons that may be impossible by experimental means and (iii) making sense of data by uncovering hidden patterns and trends. In the context of drug discovery efforts, *in silico* approaches can be used to not only help identify and prioritize classes of compounds to screen but it can also help reduce the number of compounds to be tested. Quantitative structure–activity relationship (QSAR) is ligand-based approach in computational drug design for correlating the molecular features of a chemical library with their respective bioactivity.^26–28^ QSAR has been instrumental in shedding light on the molecular basis of bioactivity of interest by learning from past bioactivity data while also being amenable to extrapolating on the bioactivity of new compounds that are foreign to the trained data set. The utilization of QSAR for the investigation of ERs had started in 1986 where Singh^29^ examined the binding affinity of 2-phenylindoles towards estrogen receptor using Kiers first-order valence molecular connectivity index. Thusfar, there exist 56 research articles reporting the QSAR modeling of ER inhibitors according to a search on Scopus for articles containing (QSAR or QSPR or “quantitative structure–activity relationship”) and (“estrogen receptor” or ERα or ERβ) as search query. A brief analysis of the existing QSAR models of ER revealed that nearly all are based on small data sets that are typically less than 50 compounds (*i.e.* belonging to the same congeneric class) while focusing on the selectivity of inhibitors against the two ER isoforms *via* classical QSAR^30–32^ and 3D-QSAR.^33,34^ However, there were only a few studies reporting the use of large data set for the QSAR modeling of ER inhibitors. Among this are the work of Gao *et al.*^35^ whose data set consisted of 463 compounds, the work of Mekenyan *et al.*^36^ reporting a data set size of 151 compounds and the work by Fang *et al.*^37^ on a set of 230 compounds.

**Fig. 2:**
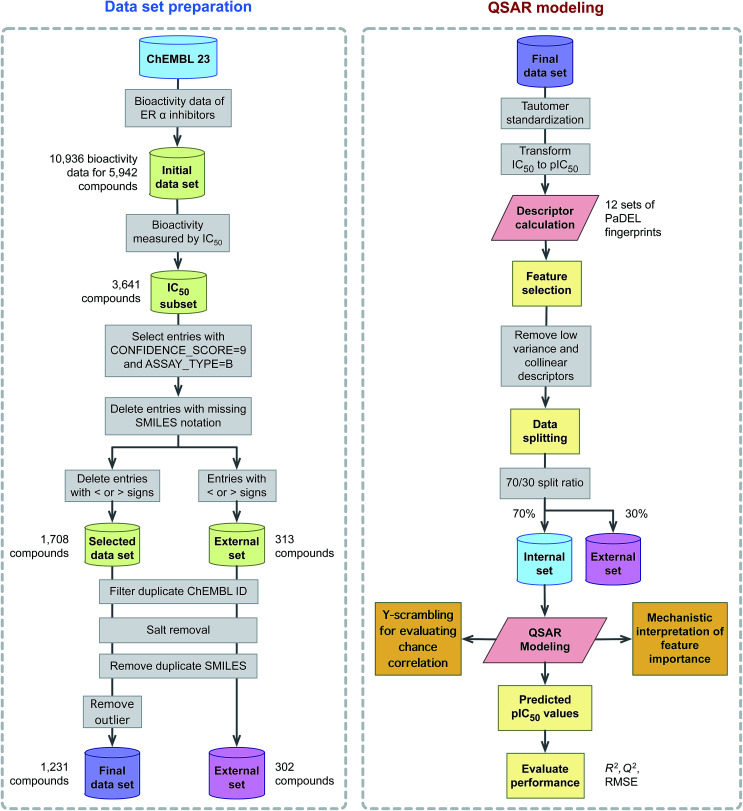
Workflow of QSAR modeling for predicting ERα inhibitory activity.

This study explores the origin of ERα inhibitory activity by discerning their underlying structure–activity relationship *via* QSAR modeling. To achieve this, interpretable and simple to compute descriptors in concomitant with interpretable learning method were employed. The effectiveness and usefulness of twelve classes of fingerprint descriptors for model construction was determined. Molecular features important for the investigated ERα inhibitory activity were revealed *via* the Gini index and their contribution were to discerned in light of previous evidences from the literature. A schematic illustration of the QSAR modeling workflow for predicting the inhibitory activity of ERα is provided in Fig. 2.

## 2 Materials and methods

### 2.1 Data collection

A data set consisting of 5809 compounds with 10 936 bioactivity data points targeting human ERα (CHEMBL206) was obtained from the ChEMBL database,^38^ version number 23. A subset of the data reporting IC_50_ as the bioactivity value was selected for further investigation and this consisted of a total of 3641 compounds. Next, entries with < or > signs were subjected to removal as external data set while entries having CONFIDENCE_SCORE equal to 9 (*i.e.* a direct single protein target is assigned) and those with ASSAY_TYPE equal to B (*i.e.* binding measurements of compounds to molecular targets as provided by *K*_i_, IC_50_ and *K*_D_ values) were selected for further use. Moreover, redundant compounds with (i) identical SMILES notation (ii) IC_50_ value greater than 2 SD (*i.e.* if less than 2 SD then the median value is used) and (iii) missing IC_50_ values were eliminated thereby further reducing the data set to 1780 compounds. After that, the SMILES notation for all entries from the data set was subjected to salt removal followed by its conversion to 2D structures using the *Chem* function of RDKit.^39^ Finally, after desalting the chemical structure, a set of 477 compounds having identical SMILES notation were removed. This resulted in the final data set consisting of 1231 compounds. It is also worthy to note that IC_50_ values were converted to pIC_50_ units (−log IC_50_) so as to afford a more uniform distribution of the data.

### 2.2 Descriptor calculation

Each compound was encoded by several sets of fingerprint descriptors computed using the PaDEL-Descriptor software.^40^ Briefly, molecular fingerprint is a widely used molecular descriptor in cheminformatics owing to their ability to capture the feature space of chemical structures. However, the performance difference existing among these different fingerprint types has been the subject of several investigations on its utilization for bioactivity modeling.^41^ Hence, this study considers 12 classes of molecular fingerprints consisting of AtomPairs 2D count, AtomPairs 2D, CDK fingerprinter, CDK extended, CDK graph only, E-state, Klekota–Roth count, Klekota–Roth, MACCS, PubChem, substructure count and substructure were used in this study. A summary of these descriptor types is provided in Table 1. Briefly, chemical structures stored in the MOL format were used as input for the calculation of fingerprints. For each compound, polar hydrogen atoms were added and tautomers were standardized prior to fingerprint calculation.

**Table 1:** Summary of 12 sets of PaDEL fingerprint descriptors employed in this study

Fingerprint class	Descriptors	Description	Reference
AtomPairs 2D count	780	Count of atom pairs at various topological distances	42
AtomPairs 2D	780	Presence of atom pairs at various topological distances	42
CDK fingerprinter	1024	Fingerprint with length of 1024 and search depth of 8	43
CDK extended	1024	Extends the fingerprinter with additional bits describing ring feature	43
CDK graph only	1024	Special version of fingerprinter not taking bond orders into account	43
E-State	79	E-State fragments	44
Klekota–Roth count	4860	Count of chemical substructures	45
Klekota–Roth	4860	Presence of chemical substructures	45
MACCS	166	Key-based fingerprint which uses 166 predefined keys	46
PubChem	881	PubChem fingerprints	
Substructure count	307	Count of SMARTS patterns for functional group classification	47
Substructure	307	Presence of SMARTS patterns for functional group classification	47

### 2.3 Data pre-processing

Prior to construction of the classification model, descriptors were subjected to mean centering and unit variance scaling as to afford comparability. Descriptors were removed if pairwise inter-correlation coefficients exceed the threshold value of 0.95 and correlation coefficient exceed the threshold value of 0.7. This resulted in reduced subsets consisting of 120, 154, 31, 951, 934, 599, 405, 452, 92, 196, 64 and 66 descriptors for AtomPairs 2D Count, AtomPairs 2D, CDK fingerprinter, CDK extended, CDK graph only, E-state, Klekota–Roth count, Klekota–Roth, MACCS, PubChem, substructure count and substructure, respectively, as summarized in Table 1.

### 2.4 Data splitting

In the construction of prediction models, the possibility of bias may arise from a single data split. In order to address this problem, Puzyn *et al.*^48^ suggested that prediction models should be constructed from *N* independent data splits. Thus, this study employs independent data splits using a split ratio of 70/30 where 70% of the entire data set was used as the internal set and the remaining 30% served as the external set. The final prediction performance was obtained by calculating the mean and standard deviation values for statistical parameters from these independent data splits.

### 2.5 Multivariate analysis

Regression models afford the prediction of a continuous response variable (*e.g.* pIC_50_) as a function of predictors (*e.g.* fingerprint descriptors) *via* the use of learning algorithms. Random forest (RF) is well-known as an ensemble machine learning technique that is capable of handling both classification and regression tasks by making use of multiple decision tree learners to collectively predict the value of a target observation.^49^ The RF model have been developed to improve the prediction performance of classification and regression trees (CART) by harnessing the power of several weak CART models.^50^ In the construction of a model, every CART is built from a fixed number of randomly selected features for tree splitting while a bootstrap technique is used for sampling from the entire data set. RF boasts several advantages: (i) resilience toward overfitting, (ii) provides built-in feature selection and (iii) relatively fast model building. RF models were constructed using the *RandomForestClassifier* function from the scikit-learn machine learning library in Python.^51^ Optimization of training parameters was performed programmatically by iteratively constructing regression models with each parameter adjustment.

### 2.6 Model assessment

One of the crucial processes in developing a QSAR model is the evaluation of the model's performance and robustness or validity of the model prior to its usage in predicting and interpreting the biological activities of compounds. Quantifying the confidence and predictive accuracy of a model provides the decision-maker with the information necessary for establishing well-informed decisions. The squared value of Pearson's correlation coefficient (*i.e. R*_Tr_^2^ and *Q*_CV_^2^ for training and cross-validated sets, respectively) and root mean squared error (RMSE) are two standard statistical parameters that are commonly used for evaluating the performance of QSAR models.

This study employs 10-fold cross-validation (10-fold CV) to evaluate the model's performance in which a data set is partitioned into 10 data subsets after which 9 subsets are used to train a model and subsequently evaluated on the held out subset (*i.e.* used as the test set). This procedure was repeated iteratively until all data subset had a chance to be held out as the test set while the remaining subsets were used as the training set for model building.

After construction of the RF model, a reduced subset of top 20 features were selected for the construction of the second RF model so as to avoid over-fitting and to satisfy the philosophical Occam's razor principle in which a simple explanation is favorable to a more complicated one where analogously a model with fewer descriptors that still afford robust level of performance is preferable to a model with significantly higher descriptors.

Furthermore, external validation (*Q*_Ext_^2^) was performed on the held out 30% external set. The reliability of QSAR models was provided by the difference of *R*^2^ and *Q*^2^ as originally proposed by Eriksson *et al.*^52^ Further rigorous test for the possibility of chance correlation was performed *via* Y-scrambling experiments in which the X–Y pairs are shuffled such that the resulting X–Y pairs are false pairs. If the resulting shuffled models afforded similar level of prediction performance with that of the original X–Y pair then it could be concluded that the model's performance is unreliable and arose by chance correlation. However, if the Y-shuffled models provided poor performance in comparison to the high performance of the original X–Y pair then it is indicative of the model's robustness. A total of 100 Y-scrambled models were computed.

### 2.7 Applicability domain analysis

The applicability domain of the QSAR model presented herein is assessed by means of the principal component analysis (PCA) bounding box approach.^53,54^ This essentially entails comparing the chemical space of compounds from the training set with those from the external set *via* PCA analysis of scores plot. This was performed using the PCA function from the *sklearn.decomposition* module from the scikit-learn machine learning library in Python.

### 2.8 Reproducible research

To afford the reproducibility of this research, the code and data used in the construction of QSAR models and analyses performed herein are provided publicly at https://github.com/chaninlab/estrogen-receptor-alpha-qsar/.

## 3 Results and discussions

The Organization for Economic Co-operation and Development (OECD) had defined a set of rules^55^ for the development of robust QSAR models as follows: (i) defined endpoint, (ii) unambiguous algorithm, (iii) defined applicability domain, (iv) evaluation of the model's predictive potential and (v) mechanistic interpretation. These OECD principles were implemented herein as to ensure the robustness of constructed QSAR models.

### 3.1 Chemical space and applicability domain analysis

An exploration of the general chemical space of the investigated data set by means of Lipinski's rule-of-five (Ro5) descriptors is provided in ESI Fig. 1† where vertical dotted lines denotes the threshold values. Moreover, the relative spread of pIC_50_ values as a function of binned descriptor values of Ro5 descriptors are shown in ESI Fig. 2.† Briefly, the Ro5 describes the drug-likeness of compounds on the basis of their molecular properties namely molecular weight (<500), octanol–water partition coefficient (log *P*; <5), the number of hydrogen bond acceptors (<10) and the number of hydrogen bond donors (>5). As useful as the Ro5 are, they have been shown to afford limited value in contributing to our understanding on the underlying principles of the target–ligand relationship (*i.e.* the affinity of the ligand toward the target) as they were strictly based on general molecular properties of the ligand. Oprea *et al.*^56^ showed that the Ro5 criteria do not serve to discriminate drugs from non-drugs in which more than 90% of the compilation of chemical reagents known as the Available Chemicals Directory were also Ro5 compliant. However, this does not negate the notion that the criteria exemplified by the Ro5 cannot be used to narrow properties that are useful for therapeutically relevant pharmacokinetic space. Moreover, Benet *et al.*^57^ has shown that QSAR model built using the Ro5 criteria could successfully predict drug disposition characteristics for drugs both meeting and not meeting the Ro5 criteria.

**Fig. 3:**
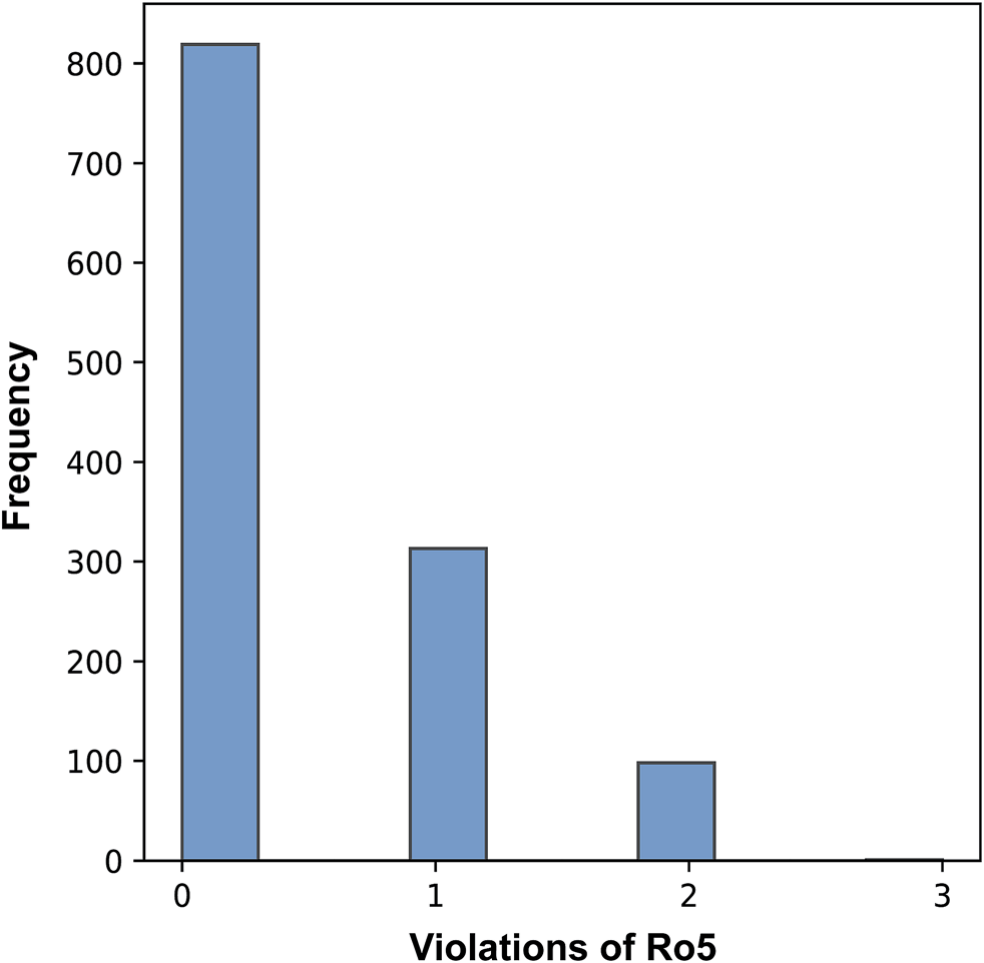
Plot of the distribution of compounds with 0 to 3 violations of the Ro5 criteria.

**Fig. 4:**
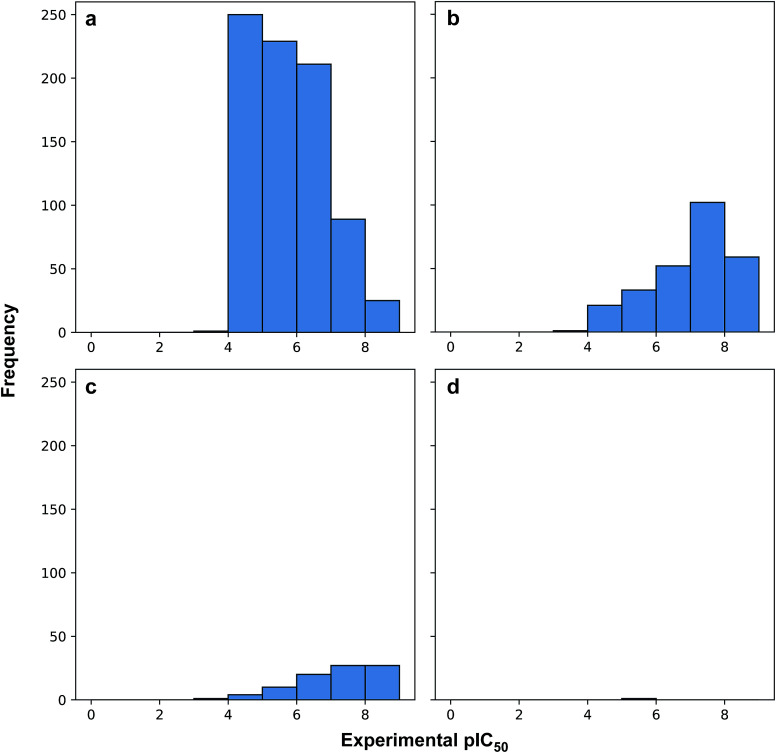
Histogram plots of the distribution of pIC_50_ values for compounds in violation of zero (a), one (b), two (c) and three (d) Ro5 descriptors.

Fig. 3 revealed that of the 1231 compounds present in the curated data set, roughly two-third of compounds had zero violation while the other one-third of compounds are distributed between one and two violations. In this latter set, approximately three-quarter fell in the one Ro5 violation spectrum with the remaining one-quarter falling within the two Ro5 violation zone. It is interesting to note that as the number of Ro5 violations increased, the bioactivity also increased (Fig. 4).

A closer look revealed that a minority proportion of compounds in violation of the Ro5 was due to the fact that it had molecular weight greater than 500 Da. On the other hand, a larger proportion of compounds in violation of the Ro5 was because they had log *P* value greater than 5. In spite of this, it should be noted that Lipinski *et al.*^58^ pointed out that compounds in violation of the Ro5 should not necessarily be removed from further consideration. In fact, efforts have been directed to soften the Ro5 ^59^ as it is well known that there are several instances where therapeutically useful drugs are in violation of several Ro5 parameters such as Atorvastatin, Lipitor, Losartan, Montelukast, Olmesartan, Telaprevir, Telmisartan, *etc.* It is worthy to note that the Ro5 should be used sparingly as general guidelines and not as strict rules so as to set loose criteria that would allow the discovery of potent drug candidates that may at first glance be removed if the Ro5 criteria was strictly followed.

**Fig. 5:**
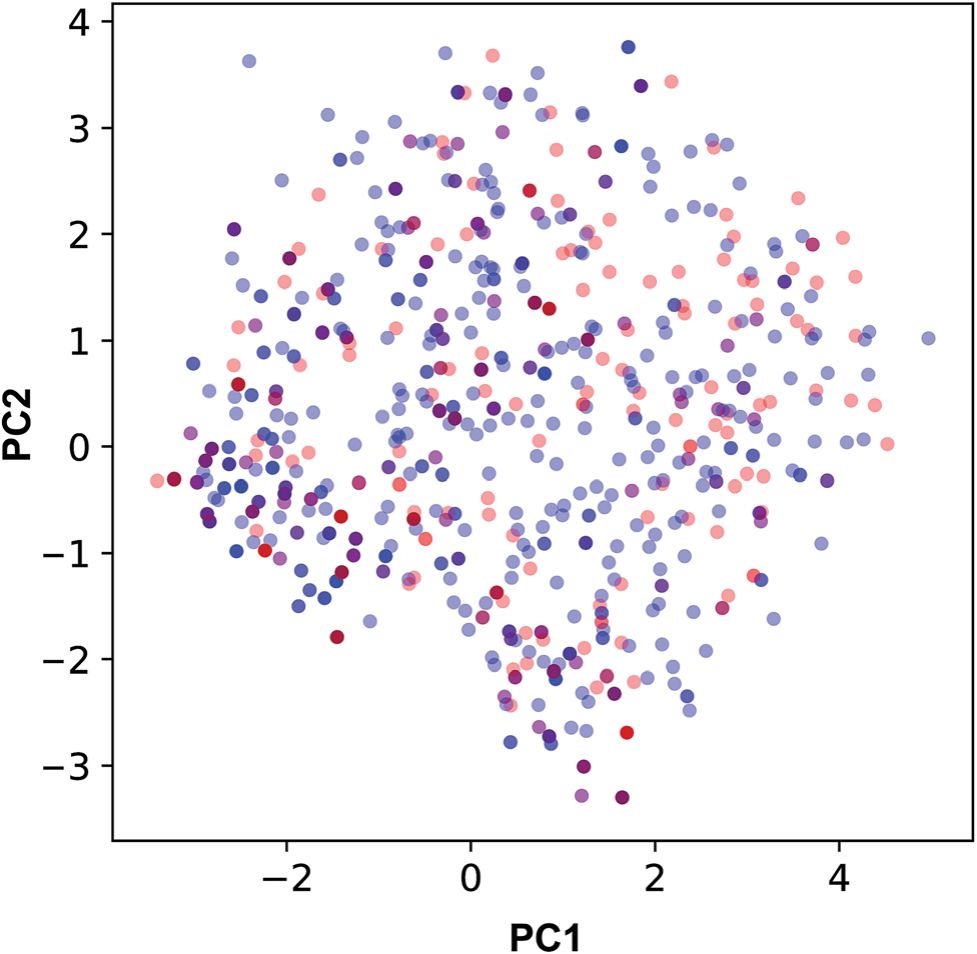
Applicability domain analysis as deduced from the PCA scores plot of compounds from internal (blue) and external (red) sets constituting 70% and 30% of the data set, respectively.

**Fig. 6:**
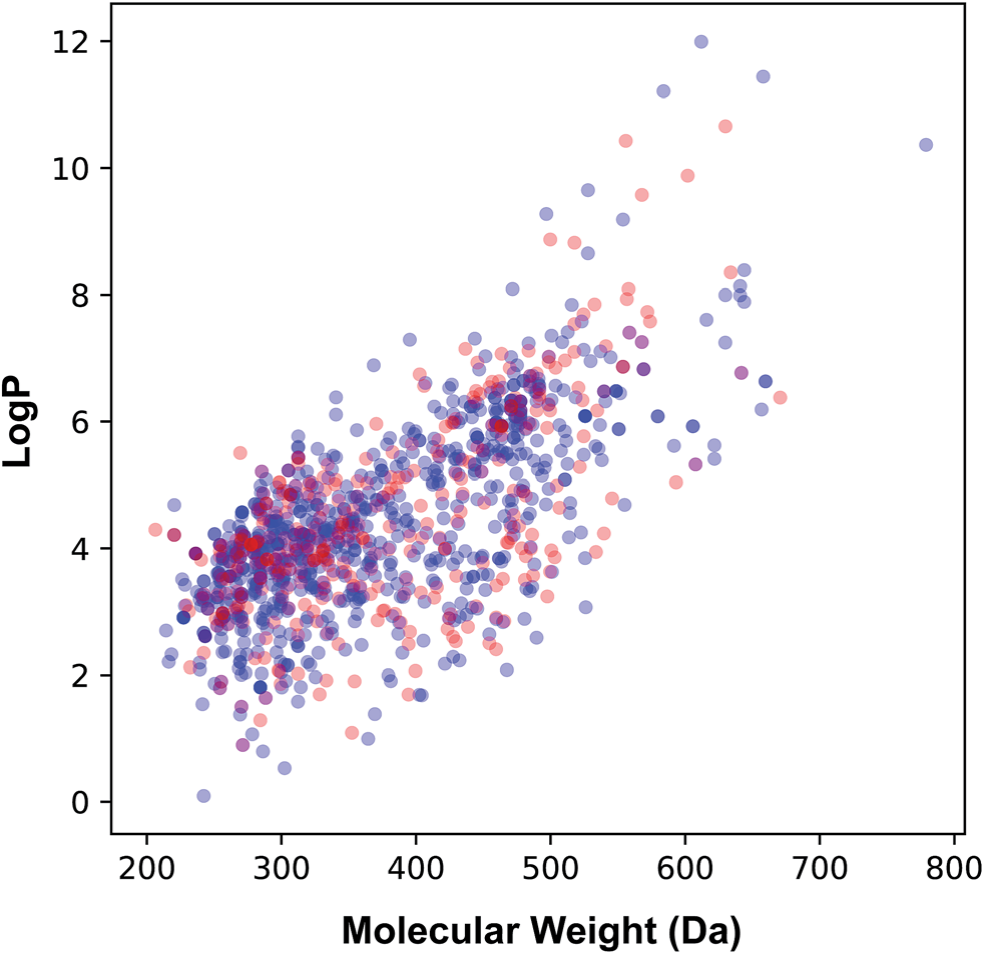
Plot of the molecular weight *versus* lipophilicity for the internal (blue) and external (red) sets that constituting 70% and 30% of the data set, respectively.

The applicability domain of the QSAR model proposed herein was assessed *via* the PCA bounding box approach in which the chemical space spanned by the training set (*i.e.* the 70% subset) is compared to that of the external set (*i.e.* the 30% subset) as shown in Fig. 5. It was found that the chemical space spanned by the external set falls within the boundaries of the chemical space of the training set and thus is also deemed to be within the applicability domain of the constructed QSAR model. Moreover, the relative chemical space spanned by compounds from internal and external sets as visualized in Fig. 6 can be seen to share a high degree of similarity as also seen from the PCA scores plot.

### 3.2 QSAR modeling

The curated data set comprising of 1231 compounds was used for the construction of QSAR models for predicting the ERα inhibitory activity of a structurally diverse compounds spanning several scaffolds. Molecular features of compounds were described by several fingerprint types. The intrapolation and generalization ability of QSAR models was examined on internal and external sets obtained from several rounds of data splits. Each of the twelve models were built using a data split ratio of 70/30 in which 70% of the data set was used as the internal set and 30% as the external set. The first data subset consisting of 70% was used for internal validation of the QSAR model (*i.e.* used as the training set as well as the cross-validation set) and its performance was consequently evaluated by *R*^2^, *Q*^2^ and RMSE. The second data subset containing 30% of the bioactivity data was utilized for external validation and their performance was assessed by *Q*^2^ and RMSE.

Models were constructed *via* the RF algorithm and their results are presented in Table 2. Assessment of the predictive performance of the model was performed according to the suggested statistical thresholds of Golbraikh and Tropsha^60,61^ in which acceptable models should have *R*^2^ > 0.6 and *Q*^2^ > 0.5.^60^ Results indicated that the two best models as judged from both internal and external validation, which consisted of AtomPairs 2D count (*R*_Tr_^2^ = 0.93, *Q*_CV_^2^ = 0.73 and RMSE_Tr_) and substructure count (*R*_Tr_^2^ = 0.94 and *Q*_CV_^2^ = 0.73). Particularly, the substructure count was selected for further investigation owing to its interpretability and fewer number of descriptor (*i.e.* 307 descriptors as compared to 780 to 4860 descriptors from the other fingerprints), which also require less computation time.

**Table 2:** Summary of predictive performance for QSAR model of ERα inhibitory activity

Fingerprint class	Training set		External set		*R*_Tr_^2^ − *Q*_Ext_^2^
	*R*_Tr_^2^	RMSE_Tr_	*Q*_Ext_^2^	RMSE_Ext_	
AtomPairs 2D count	0.93	0.38	0.73	0.53	0.20
AtomPairs 2D	0.85	0.54	0.68	0.62	0.17
CDK fingerprinter	0.87	0.51	0.71	0.56	0.16
CDK extended	0.84	0.55	0.67	0.65	0.18
CDK graph only	0.81	0.60	0.70	0.58	0.11
E-state	0.80	0.63	0.64	0.71	0.16
Klekota–Roth count	0.91	0.41	0.72	0.54	0.19
Klekota–Roth	0.82	0.60	0.70	0.59	0.12
MACCS	0.86	0.52	0.71	0.58	0.15
PubChem	0.84	0.57	0.71	0.56	0.12
Substructure count	0.94	0.34	0.73	0.52	0.21
Substructure	0.87	0.51	0.68	0.63	0.19

**Fig. 7:**
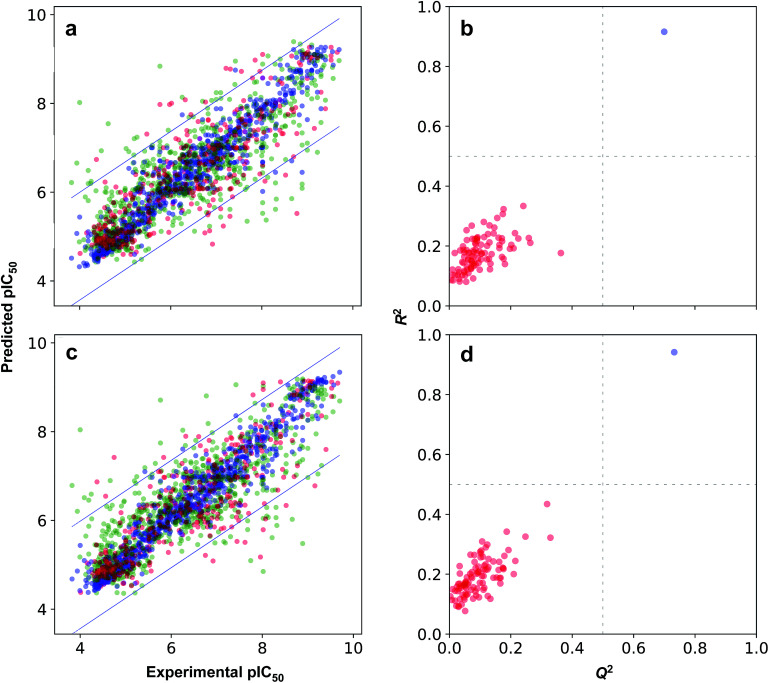
Plot of the predicted *versus* the experimental pIC_50_ values (a and c) and plot of Y-scrambled models (b and d). Models were built using AtomPairs 2D Count (a and b) and substructure count (c and d) fingerprints. For plots in the left panel, data samples from training, cross-validated and external sets are shown in green, red and blue colors, respectively, while the 2 SD line are shown in blue. For plots in the right panel, Y-scrambled and actual models are shown in red and blue colors, respectively.

The possibility for chance correlation can be assessed from the *R*^2^–*Q*^2^ margin as described by Eriksson^52^ where values <0.2–0.3 are indicative of predictive and reliable models while values >0.2–0.3 suggests possible chance correlation or the presence of outliers in the data set. Furthermore, from observation of the *R*_Tr_^2^ − *Q*_Ext_^2^ margin, it is revealed that differences were negligible with values not greater than 0.2. Fig. 7a and b shows the scatter plots of experimental *versus* predicted pIC_50_ values. As for the threshold value for RMSE, which is rather difficult to establish, but generally models with higher RMSE values can be considered to afford sub-optimal prediction. Such high RMSE value may be due to the presence of a small number of outlying compounds that give rise to high error predictions.^62^ Furthermore, the inherent variability of experimental assays in concomitant with the diversity of chemotypes present in the data set are also expected to directly give rise to prediction error.^63,64^

Moreover, the proposed models were further subjected to stringent test to evaluate the possibility of chance correlation by carrying out Y-scrambling experiments. Briefly, this encompassed the shuffling of the X block of descriptors with that of its corresponding Y label such that the shuffled data set have false X–Y pairs whereas the original data set had true X–Y pairs. Fig. 7b and c shows the results from the Y-scrambling experiment and it can be seen that original models (*i.e.* denoted by blue circles) for all fingerprint class are found to be located at the upper right quadrant thereby suggesting robust models in accordance with the threshold of Golbraikh and Tropsha.^60^ On the other hand, Y-scrambled models (*i.e.* represented by red circles) were found to be lying within the boundaries of the lower left quadrant, which is indicative of their poor performance.

### 3.3 Additional external validation

To further evaluate the model performance, additional external sets consisting of qualitative bioactivity class labels (*i.e.* IC_50_ values having < or > signs) were used. This external set was pre-processed in the same manner as that of the internal set and the aforementioned top 20 features were used as descriptors. The first external set is comprised of 283 compounds with > sign in the bioactivity class label and the internal set was found to afford an accuracy of 0.88 for this external set (*i.e.* predicted values had higher value than the specified value in the bioactivity class label). In contrast, the second external set in which compounds having < sign in the bioactivity class label could produce a rather low accuracy of 0.16. A closer analysis revealed that compounds in the former external set were experimentally evaluated using bioactivity assay formats also found in the training set whereas the bioactivity assay formats for compounds in the second external set was not found in the respective training set. Thus, it could be rationalized that compounds in the first external set (*i.e.* having > sign in the bioactivity class label) were in the applicability domain as those used to train the model whereas those in the second external set (*i.e.* having < sign in the bioactivity class label) were outside the applicability domain of the trained model owing to inherent differences in the assaying methods of the training set and that of this second external set as well as the fact that compounds in this set are less presented in the training set.

### 3.4 Mechanistic interpretation of important features

**Fig. 8:**
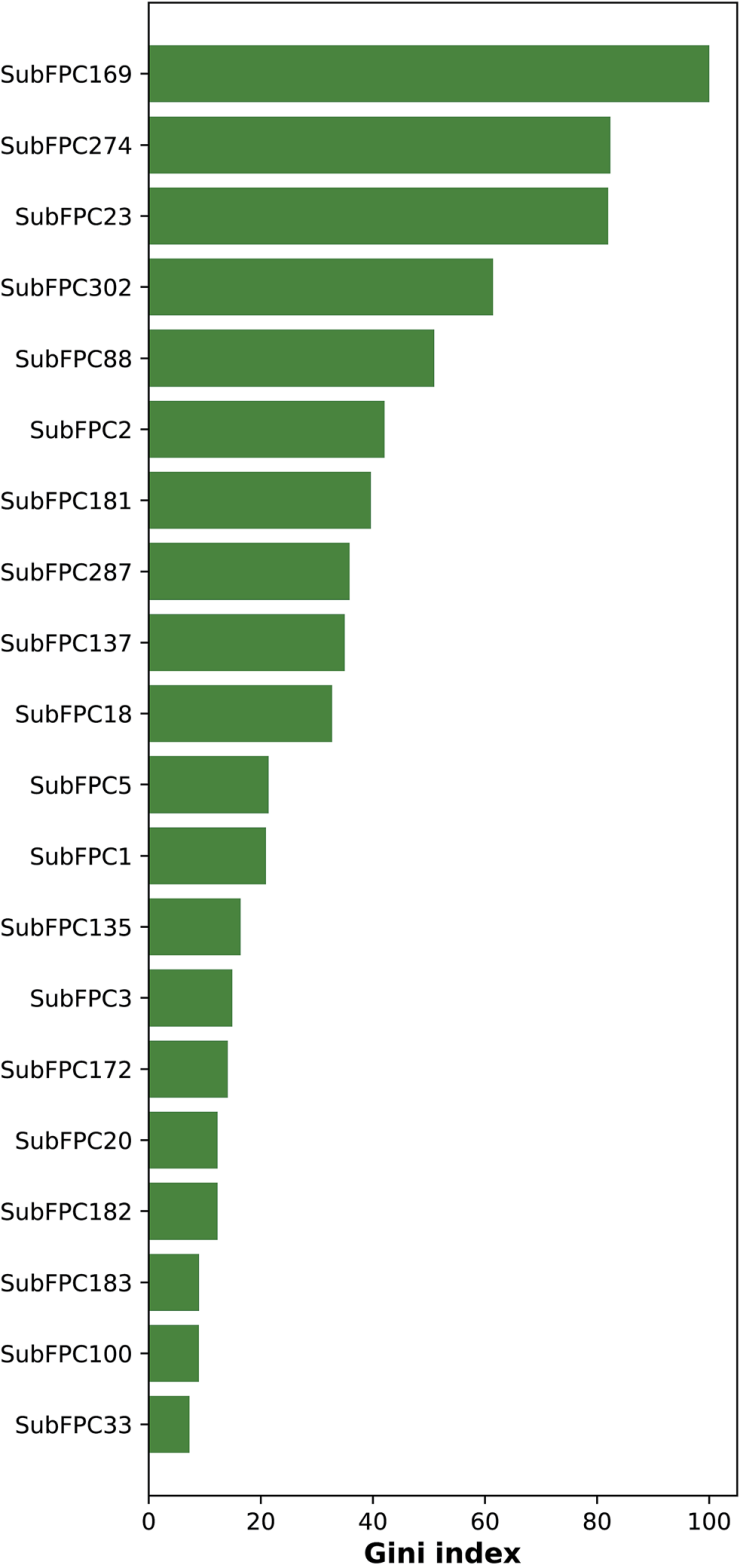
Plot of the mean decrease of Gini index for rationalizing the feature importance.

Important features that are important for the investigated bioactivity could be deduced from the constructed QSAR models by analyzing the Gini index. Fig. 8 ranks these important features by displaying the mean decrease of the Gini index. Table 3 lists the top-ranking substructure count descriptors along with their respective description.

**Table 3:** List of the top 10 substructure count and their corresponding description

Fingerprint class	Description
SubFPC169	Phenol
SubFPC307	Chiral center specified
SubFPC274	Aromatic
SubFPC295	C ONS bond
SubFPC23	Amine
SubFPC300	1,3-Tautomerizable
SubFPC301	1,5-Tautomerizable
SubFPC26	Tertiary aliphatic amine
SubFPC88	Carboxylic acid derivative
SubFPC302	Rotatable bond

As can be seen in Fig. 8, the top ranking feature is phenol (SubFPC169), which contains a 1,2-benzenediol moiety and belongs to the class of organic compounds known as catechols. Catechols are secondary metabolites found in many plants that have been shown to confer numerous bioactivities.

The second top-ranked feature is the aromatic (SubFPC274) descriptor, which is a ubiquitous substructure that plays an important structural role as scaffolds of compounds as well as functional moieties that mediates π–π stacking interaction. Differences in the number and type of atoms in the aromatic rings of molecules can present various development concerns such as aqueous solubility, lipophilicity, serum albumin binding, cytochrome P450 inhibition and hERG inhibition.^65^

The third top-ranked feature is amine (SubFPC23) which present in amino acids are used to form bonds that are essential for their electron donation property.

The bioavailability of a drug-like molecule is related to its rotatable bond (SubFPC302), the fourth important feature, number where drug-like compounds have 10 or fewer rotatable bonds. Although, this is not absolute as some effective inhibitors carry more than 12 rotatable bonds.^66^ In recent years, many highly potent molecules carrying more than 10 rotatable bonds are still administered through the oral route with some modifications to their dosage forms.

The fifth and sixth important features are amine (SubFPC88) and secondary carbon (SubFPC2), respectively. Carboxylic acid is a common functional group found in the pharmacophore of diverse classes of therapeutic agents.^67^ Currently, a large number (>450) of carboxylic acid-containing drugs have been marketed worldwide. The secondary carbon, which is attached to two other carbons, is also a common component in the structure of some anti-cancer agents.^68^

The seventh and eighth important features are hetero N nonbasic (SubFPC181) and conjugated double bond (SubFPC287), respectively. Hetero N nonbasic can be defined as an aromatic nitrogen atom having two further total connections or an aromatic nitrogen atom affording a charge of +1 with three further total connections. Therefore both features are essential for anticancer activity in compound structure.^69,70^ In a conjugated double bond, the double bonds are separated by two or more methylene groups and can react with nucleophiles in a similar fashion as the aromatic ring (*i.e.* withdrawing electrons from electronegative atoms).

The ninth and tenth important substructures are vinylogous ester (SubFPC137) and alkyl aryl ether (SubFPC18), respectively. These two functional groups have been found in several breast cancer drugs.^71,72^ Alkyl aryl ether is also a key substructure of Tamoxifen, which is a selective estrogen receptor modulator as well being one of the oldest and most-prescribed FDA-approved drug for hormonal therapy.^73^

### 3.5 Structural analysis of important features

**Fig. 9:**
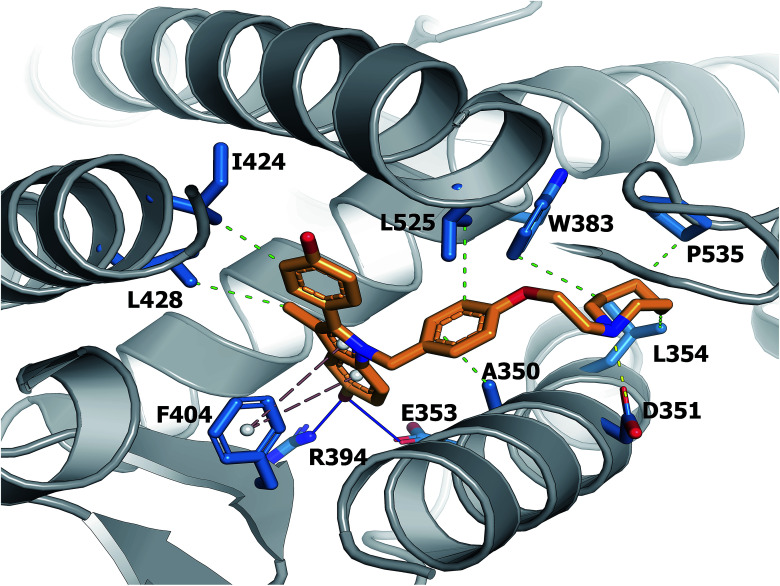
Binding pocket of the ligand-binding domain of ERα in complex with CHEMBL304552 (PDB id: 1SJ0). Helices and sheets are depicted in gray color, the ligand is represented in orange colored sticks while its interacting residues are colored blue. Important interactions are indicated by colored dashed lines as follows: brown, hydrophobic interactions; yellow, salt bridge; green, π-stacking interaction (parallel).

A large set of 240 crystal structures of human ERα ligand-binding domain was retrieved from RCSB Protein Data Bank. An analysis of the active site of these structures revealed that most afforded hydrogen bonding to Arg394, His524 and Glu353. A closer observation of the ligand-binding domain of ERα (PDB id: 1SJ0) as shown in Fig. 9 revealed that Glu353 and Arg394 are engaged in hydrogen bonding with the oxygen atom from the ligand's phenol moiety. Important features as obtained from the QSAR model corroborate the aforementioned molecular interaction. Particularly, this includes phenol (SubFPC169), C–ONS bond (SubFPC295) and carboxylic acid derivative (SubFPC88). Moreover, π–π interaction with Phe336 were also found to be prevalent amongst the molecular interaction with ligands. This is well supported by the aromatic (SubFPC274) descriptor from the QSAR model.

## 4 Conclusion

Molecular fingerprints is a robust descriptor type with immense utility in cheminformatics and computer-aided drug design owing to its information-rich description on the structural details of investigated compounds. The advantage of these descriptors is that they can be rapidly generated in a high-throughput fashion while also affording robust performance and interpretability. In this work, we elucidate the origin of ERα inhibitory activity *via* QSAR models based on molecular fingerprints. In this study, we performed a comparative evaluation of the classification performance afforded by twelve fingerprint types using the ensemble learning approach based on random forest. Important features contributing to ERα inhibitory activity were deduced from the Gini index of top-ranking substructure fingerprints. It was found that 1,2-diphenol, primary aliphatic amine, quaternary aliphatic ammonium, carbothioic acid, acyliodide, diaryl ether bond, tertiary carbon, vinylogous amide, conjugated triple bond and nitrite were important substructures for the observed ERα inhibitory activity. Thus, the QSAR model proposed herein has great utility as a high-throughput platform that can be used to screen large chemical libraries for identifying promising hit compounds for further experimental validation. Moreover, the molecular insights gained are also useful as guidelines for the design of robust ERα inhibitors.

## Author's contributions

CN conceived and designed the study. NS coded the QSAR modeling workflow using the Python programming language. NS and CN analyzed the contribution of molecular features on ERα inhibitory activity. NS, AAM and CN prepared figures of the protein structure of ERα and reviewed the literature on the structural basis of its inhibition. NS, LP, WS, AAM, AW, TT and CN took part in discussion and analysis of results. NS and CN drafted the manuscript. CN vetted and finalized the manuscript. All authors read and approved the final manuscript.

## Conflicts of interest

There are no conflicts to declare.

## Supplementary Material

RA-008-C7RA10979B-s001
