# Supplementary material for: Probing the origin of estrogen receptor alpha inhibition via large-scale QSAR study
Source: RSC Adv. 2018 Mar 27;8(21):11344–56. doi: 10.1039/c7ra10979b (PMC9079045; doi:10.1039/c7ra10979b)
Supplement: RA-008-C7RA10979B-s001 [file RA-008-C7RA10979B-s001.pdf]

Electronic Supplementary Material (ESI) for RSC Advances.
This journal is © The Royal Society of Chemistry 2018

Supplementary Information

Probing the origin of estrogen receptor alpha inhibition via large-scale QSAR study

Naravut Suvannang[a], Likit Preeyanon[b], Aijaz Ahmad Malik[a], Nalini Schaduangrat[a], Watshara Shoombuatong[a], Apilak Worachartcheewan[b], Tanawut Tantimongcolwat[c], and Chanin Nantasenamat[*1]

[a] *Center of Data Mining and Biomedical Informatics, Faculty of Medical Technology, Mahidol University, Bangkok 10700, Thailand*

[b] *Department of Community Medical Technology, Faculty of Medical Technology, Mahidol University, Bangkok 10700, Thailand*

[c] *Center for Research and Innovation, Faculty of Medical Technology, Mahidol University, Bangkok 10700, Thailand*

[*] Corresponding author. E-mail: chanin.nan@mahidol.edu

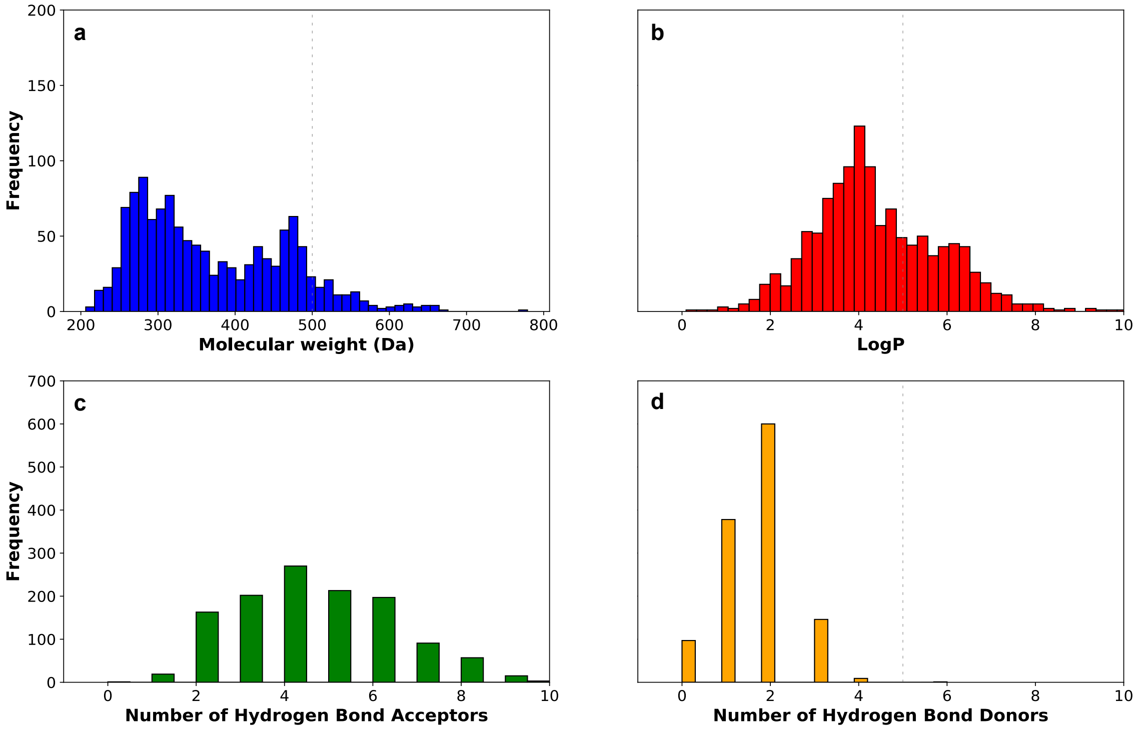

Supplementary Figure 1. Histogram plots of frequency count of Ro5 descriptors for ERα inhibitors. This consisted of molecular weight (a), LogP (b), number of hydrogen bond acceptors (c) and number of hydrogen bond donors (d).

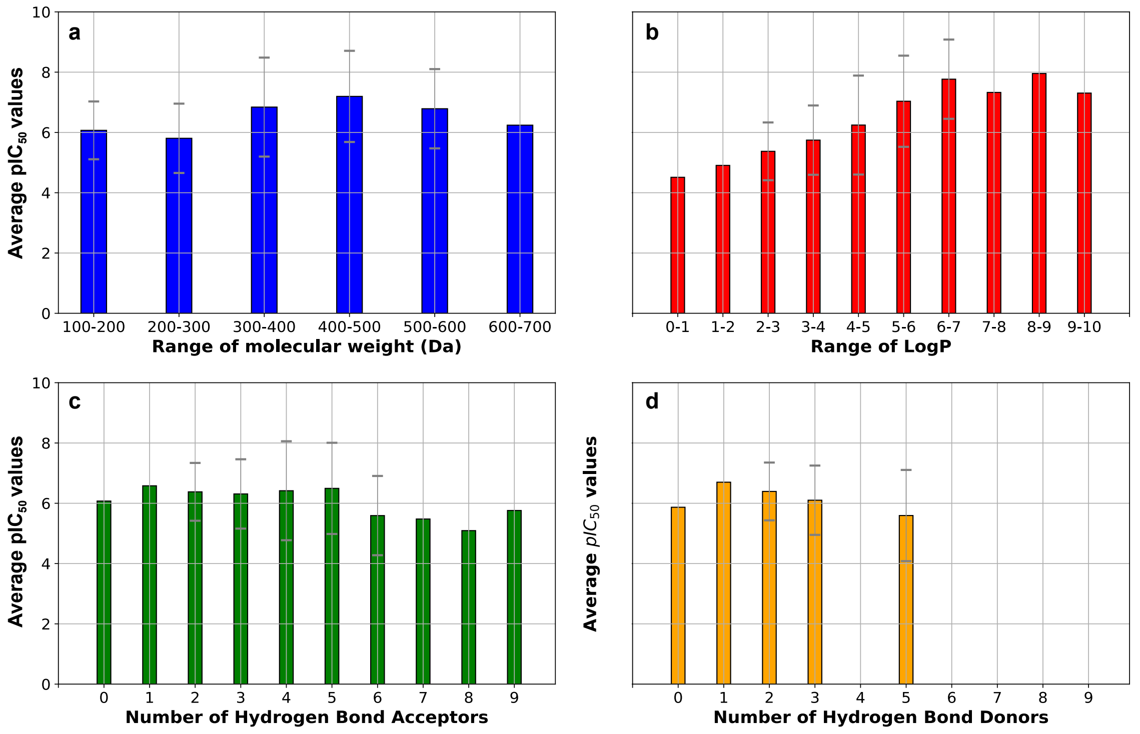

Supplementary Figure 2. Histogram plots of mean pIC_{50} values for Ro5 descriptors for ERα inhibitors. This consisted of molecular weight (a), LogP (b), number of hydrogen bond acceptors (c) and number of hydrogen bond donors (d).

Supplementary Table 1

Summary of predictive performance for QSAR model of ERα inhibitory activity.

Fingerprint class	Training set		10-fold CV set		External set		$R^2 - Q^2_{cv}$	$Q^2 - Q^2_{ext}$
	R^2_{Tr}	$RMSE_{Tr}$	Q^2_{CV}	$RMSE_{CV}$	Q^2_{Ext}	$RMSE_{Ext}$		
AtomPairs 2D count	0.93	0.38	0.73	0.72	0.73	0.53	0.20	0.003
AtomPairs 2D	0.85	0.54	0.67	0.80	0.68	0.62	0.18	-0.01
CDK fingerprinter	0.87	0.51	0.68	0.78	0.71	0.56	0.19	-0.03
CDK extended	0.84	0.55	0.69	0.78	0.67	0.65	0.16	0.02
CDK graph only	0.81	0.60	0.66	0.81	0.70	0.58	0.15	-0.04
E-State	0.80	0.63	0.66	0.82	0.64	0.71	0.14	0.02
Klekota-Roth count	0.91	0.41	0.71	0.75	0.72	0.54	0.20	-0.01
Klekota-Roth	0.82	0.60	0.65	0.82	0.70	0.59	0.16	-0.04
MACCS	0.86	0.52	0.73	0.72	0.71	0.58	0.13	0.03
PubChem	0.84	0.57	0.70	0.76	0.71	0.56	0.13	-0.01
Substructure count	0.94	0.34	0.73	0.72	0.73	0.52	0.21	-0.004
Substructure	0.87	0.51	0.69	0.77	0.68	0.63	0.18	0.02
